# Subcutaneous infusion of kisspeptin‐54 stimulates gonadotrophin release in women and the response correlates with basal oestradiol levels

**DOI:** 10.1111/cen.12977

**Published:** 2015-12-17

**Authors:** Shakunthala Narayanaswamy, Channa N. Jayasena, Noel Ng, Risheka Ratnasabapathy, Julia K. Prague, Deborah Papadopoulou, Ali Abbara, Alexander N. Comninos, Paul Bassett, Stephen R. Bloom, Johannes D. Veldhuis, Waljit S. Dhillo

**Affiliations:** ^1^ Section of Investigative Medicine Imperial College LondonHammersmith HospitalLondonUK; ^2^ Statsconsultancy LtdBucksUK; ^3^ Endocrine Research UnitCenter for Translational Science ActivitiesMayo ClinicRochesterMinnesotaUSA

## Abstract

**Background and objective:**

Kisspeptin stimulates hypothalamic GnRH secretion resulting in gonadotrophin release and has potential as a future therapeutic. Chronic subcutaneous infusion of kisspeptin via a pump (similar to an insulin pump) may provide an alternative route of administration in the future. We investigated for the first time in humans, the gonadotrophin response to subcutaneous (SC) infusions of kisspeptin‐54 in healthy women. Women are markedly more responsive to exogenous kisspeptin in the late follicular phase preovulation when oestradiol levels are naturally high. Therefore, we further investigated whether there was a correlation between baseline oestradiol levels and LH response to kisspeptin.

**Design and patients:**

A prospective, single‐blinded placebo‐controlled study. Healthy women (*n* = 4) received an 8‐h SC infusion of kisspeptin‐54 0·1, 0·3 or 1·0 nmol/kg/h or saline in the early follicular phase of 4 separate menstrual cycles. Gonadotrophins and oestradiol were measured every 10 min during the infusions.

**Results:**

SC infusion of kisspeptin‐54 increased LH and FSH. The LH response to SC infusion of kisspeptin‐54 (0·3 and 1·0 nmol/kg/h) positively correlated with baseline oestradiol levels (*P* < 0·001). Further statistical analyses showed that in the 1·0 nmol/kg/h group, a 100pmol/l rise in baseline oestradiol was associated with a 1·0 IU/l increase in LH.

**Conclusions:**

Kisspeptin administered via a SC infusion could be a viable future therapeutic route of administration for patients with infertility. Baseline oestradiol levels may be an important determinant of the gonadotrophin response to kisspeptin treatment in women and should be taken into consideration when evaluating gonadotrophin response.

## Introduction

Kisspeptin is a recently discovered hypothalamic hormone that is crucial for puberty and reproduction.^1^, ^2^ Kisspeptin stimulates the hypothalamo‐pituitary–gonadal (HPG) axis by acting on kisspeptin receptors on GnRH neurons resulting in GnRH release 3, 4 and thus is emerging as a novel therapeutic agent. Current treatments for infertility include GnRH and gonadotrophins^5^; however, there is potential for overstimulation of the HPG axis.^6^ Response to kisspeptin may be more physiological than direct pituitary stimulation as the effects of kisspeptin are limited by a patient's endogenous GnRH reserve.^4^, ^7^

Kisspeptin peptides which stimulate gonadotrophin release have a short half‐life (27·8 min for kisspeptin‐54 and 4 min for kisspeptin‐10).^8^, ^9^ A number of recent studies have administered kisspeptin to humans either intravenously (IV) or subcutaneously (SC).^10^ However, if kisspeptin is to be administered at home as a potential therapeutic to patients, then either SC bolus injections or SC infusion via a pump are possible routes of administration. Insulin pumps are now used routinely for the treatment of patients with type 1 diabetes.^11^ Therefore, pump administration (i.e. SC infusion) of kisspeptin may be an alternative treatment modality as it could be given for prolonged periods and be managed at home by the patient. However, no previous study has investigated the gonadotrophin response to SC infusion of kisspeptin in humans.

Administration of kisspeptin continuously via a SC pump could result in desensitization of kisspeptin receptors (tachyphylaxis) and hence a reduction in gonadotrophins. Twice‐daily injections of kisspeptin‐54 to women with hypothalamic amenorrhoea resulted in tachyphylaxis.^12^ However, intravenous infusions of kisspeptin‐54 resulted in a rise in LH pulsatility over the 8‐h infusion period.^13^ Thus, using smaller doses of kisspeptin administered over a prolonged period, this may be effective in achieving a sustained and meaningful rise in gonadotrophins but avoiding tachyphylaxis.

To evaluate this route of administration further, this clinical study investigated for the first time in humans the effect of SC infusions of kisspeptin‐54 on gonadotrophin response in the early follicular phase of the menstrual cycle.

Interestingly, women are markedly more responsive to kisspeptin during the preovulatory phase of the menstrual cycle compared with the early follicular phase.^9^, ^14^ High oestradiol levels are observed in the preovulatory phase compared with the early follicular phase of the menstrual cycle, and this could be a contributing factor to the differences seen in gonadotrophin response to exogenous kisspeptin in different phases of the menstrual cycle in women. Studies *in vitro* and *in vivo* have shown that GnRH stimulation by kisspeptin in the presence of oestradiol is important and lack of oestradiol reduces the secretion of kisspeptin‐induced GnRH and subsequent gonadotrophins.^15^, ^16^, ^17^ George *et al*. showed that postmenopausal women were much more responsive to IV kisspeptin‐10 than those taking sex steroid contraception or those in the follicular phase of the menstrual cycle, indicating that perhaps there is a difference in women with cyclically higher or lower sex steroids compared with exogenous sex steroids or postmenopausal low sex steroids.^18^

Chan *et al*. showed that women are least responsive to kisspeptin in the early follicular phase when oestradiol is at its lowest and despite increasing the dose of kisspeptin the LH response did not alter.^14^ We proposed to further examine the role of oestradiol in the LH response to kisspeptin by investigating whether a participant's individual baseline oestradiol level correlated with their LH response to kisspeptin administration.

## Methods

### Ethics

Ethical approval was granted by the Hammersmith, Queen Charlotte's and Chelsea Hospitals Research Ethics Committee, London. The study was performed in accordance with the Declaration of Helsinki.

### Subjects

Healthy female volunteers aged between 18 and 38 years old were recruited from local newspaper advertisements. They underwent a detailed medical evaluation as part of the prestudy screening to assess their suitability. The screening visit involved obtaining informed consent, a medical history, medication history, clinical examination, electrocardiogram and blood tests to test the following: full blood count, renal profile, liver profile, bone profile, random glucose, thyroid profile, PRL, LH, FSH, oestradiol, testosterone and SHBG. Participants were recruited who fulfilled the following criteria: regular menstrual cycles, no known medical problems or allergies, no medication use, no oral contraceptive use in the preceding 4 months, no recreational drug use and no previous research study participation or blood donation in the last 3 months. Clinical examination, electrocardiogram and blood testing were all normal. Four participants were recruited into the study. Their ages, weight, BMI, mean baseline oestradiol and mean menstrual cycle length are given in Table 1.

**Table 1:** Characteristics of participants. The demographics of the participants are shown. The weight and baseline oestradiol levels of each participant from their 4 study visits were averaged and are shown below as mean weight and mean baseline oestradiol. All mean results are presented as data ± SEM

Participant no.	Age (years)	Mean weight (kg)	Height (cm)	BMI	Mean baseline LH (IU/l)	Mean baseline FSH (IU/l)	Mean baseline oestradiol (pmol/l)	Mean menstrual cycle length (days)
1	34·92	64·6	166	23·4	5·69 ± 0·64	2·53 ± 0·22	580·9 ± 132·7	32·3 ± 1·0
2	37·75	65·4	175	21·4	2·10 ± 0·61	4·32 ± 0·76	90·3 ± 25·5	26·6 ± 1·9
3	20·58	67·4	170	23·3	5·88 ± 0·88	6·20 ± 0·91	208·6 ± 35·7	24·8 ± 0·9
4	22·17	74·6	178	23·5	4·88 ± 0·70	4·64 ± 0·33	115·5 ± 21·9	36 ± 0·4
Mean	28·86 ± 4·7	68·0 ± 2·3	172·3 ± 2·6	22·9 ± 0·5	4·64 ± 0·51	4·42 ± 0·44	249·0 ± 59·8	29·9 ± 2·8

#### Protocol

The subjects were blinded but not the investigators. The studies were conducted during the early follicular phase (day 2–6) of the subject's menstrual cycles. Each participant attended for a total of four study visits (each study day was carried out in a separate menstrual cycle) and received a different treatment at each visit (saline or kisspeptin‐54 at one of three doses tested) Subjects were asked to refrain from strenuous exercise, sexual activity and alcohol consumption for the 24‐h period preceding each study visit.

**Figure 1:**
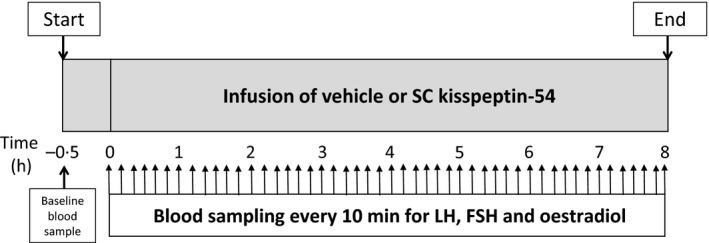
Study protocol diagram. Healthy women (*n* = 4) received a single 8‐h infusion of saline or SC kisspeptin‐54 0·1, 0·3 or 1·0 nmol/kg/h in the early follicular phase (day 2–6 of the menstrual cycle) of four separate menstrual cycles in random order. Each participant underwent a baseline blood sample 30 min prior to the kisspeptin infusion or vehicle. The infusion was started at *t* = 0 h and continued for 8‐h. Blood samples were taken every 10 min (upward arrows) for the duration of the 8‐h study to measure LH, FSH and oestradiol.

Studies were conducted in our clinical investigation unit, and Fig. 1 shows a summary of the protocol for each study visit. Subjects arrived in the morning and were cannulated in both forearms and asked to lie supine. Baseline blood sampling was performed and 30 min later an infusion of vehicle (0·9% saline) or SC kisspeptin‐54 was started and continued for 8‐h. Ten minutely blood sampling was then commenced for the duration of the 8‐h infusion until 480 min to measure gonadotrophins and oestradiol. Blood samples were collected in plain serum vacutainer tubes and were allowed to clot prior to centrifugation and separation of the serum. The samples were centrifuged at 1350 ***g*** for 10 min, separated and then frozen at −20° until analysed.

Three doses of kisspeptin‐54 were investigated: 0·1, 0·3 and 1·0 nmol/kg/h. The doses chosen have been previously shown to cause a rise in LH in healthy women following a single SC injection.^19^ Kisspeptin was administered subcutaneously into the abdomen, and the infusion rate of peptide was doubled in the first 30 min in order to achieve steady‐state plasma levels during the infusion period.^20^ Therefore, total doses of 0·85, 2·55 or 8·50 nmol/kg peptide were administered during 8‐h infusions with maintenance administration rates of 0·10, 0·30 or 1·00 nmol/kg/h, respectively. Participants had their blood pressure and pulse recorded on arrival and at regular intervals throughout the study.

#### Peptide

Human kisspeptin‐54 was synthesized by Bachem AG (Liverpool, UK) and further purified and tested as previously described.^8^, ^9^ Vials of freeze‐dried kisspeptin‐54 were stored at −20° and then reconstituted with 0·9% saline. The rate of infusion was calculated as per the weight. A set amount of the peptide solution from the reconstituted vial was transferred into the 50‐ml syringe containing 0·9% saline.

#### Analysis

Frozen samples were defrosted and analysed for measurement of LH, FSH and oestradiol using automated chemiluminescent immunoassays (Abbott Diagnostics, Maidenhead, UK). Reference ranges for females were as follows: LH 2–10 IU/l (follicular), FSH 1·5–8 IU/l (follicular and luteal) and oestradiol <300pmol/l (early follicular). The respective intra‐assay and interassay coefficients of variation for each assay were as follows: 4·1 and 3·4% (LH); 4·1 and 3·0% (FSH); 3·3% and 3·0% (oestradiol). Analytical sensitivities were as follows: 0·5 IU/l (LH), 0·05 IU/l (FSH); 37 pmol/l (oestradiol).

#### Data analysis/statistical analysis

The data were analysed by a statistician, P.B. To allow for the repeat measurements over time and multiple comparisons between groups, the analysis was performed using multilevel linear regression with Bonferroni adjustment. Linear regression analysis was also used to investigate any correlation between baseline oestradiol levels and LH response to kisspeptin‐54 at the different doses. In all cases, *P* < 0·05 was considered statistically significant and data are presented as mean ± SEM. J.D.V. used a previously described, blinded deconvolution method with 93% sensitivity and specificity to analyse LH pulsatility.^21^

## Results

Table 1 shows the participant's characteristics; mean age 28·86 ± 4·7 years and mean BMI 22·9 ± 0·5. No participants reported any adverse effects. There were no changes in heart rate or blood pressure during the studies (results not shown).

### SC infusion

**Figure 2:**
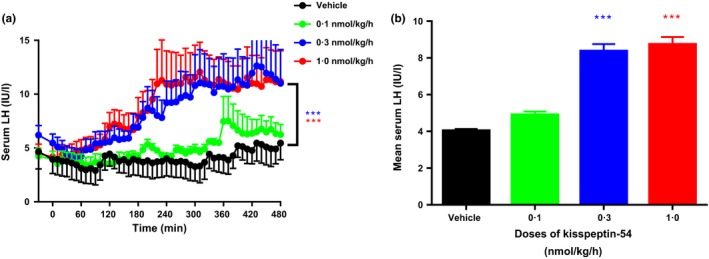
Effects on serum LH during vehicle and SC infusions of kisspeptin‐54 in healthy women during the early follicular phase of the menstrual cycle. Collated results from all participants in response to 8‐h infusions of vehicle and SC kisspeptin‐54 at 0·1, 0·3 and 1·0 nmol/kg/h doses. (a) shows the mean serum LH results for each time point (from all 4 participants) during the 8‐h study presented as a time profile. (b) is a graphical representation of the mean LH over the study period as shown in (a). Black line and bar, vehicle; green line and bar, kisspeptin‐54 0·1 nmol/kg/h; blue line and bar, kisspeptin‐54 0·3 nmol/kg/h; red line and bar, kisspeptin‐54 1·0 nmol/kg/h. Data are mean ± SEM. *N* = 4/group. Blue***, *P* < 0·001 for SC kisspeptin‐54 0·3 nmol/kg/h *vs* vehicle; red ***, *P* < 0·001 for SC kisspeptin‐54 1·0 nmol/kg/h *vs* vehicle.

At all doses tested in this study, kisspeptin‐54 caused a rise in serum LH. Fig. 2a shows a time profile of the 8‐h study, presenting the mean LH results at each time point from the four participants receiving the same treatment (vehicle or SC kisspeptin‐54 0·1, 0·3, 1·0 nmol/kg/h doses). Both vehicle and the 0·1 nmol/kg/h group had slight increases in LH values over time (*P* = 0·32, 0·1 nmol/kg/h *vs* vehicle), whereas the increase in LH was much greater for the 0·3 and 1·0 nmol/kg/h groups and this was found to be highly statistically significant when compared with vehicle for both the 0·3 and 1·0 nmol/kg/h groups (*P* < 0·001).

Fig. 2b shows a graphical representation of the mean LH over the study period from the data presented in Fig. 2a.

**Figure 3:**
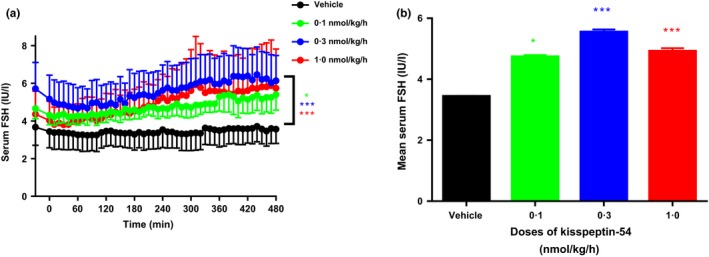
Effects on serum FSH during infusions of vehicle and SC kisspeptin‐54 in healthy women during the early follicular phase of the menstrual cycle. Mean serum FSH was collated from all participants after an 8‐h infusion of either vehicle or SC kisspeptin‐54 at 0·1, 0·3 and 1·0 nmol/kg/h doses. (a) shows the mean serum FSH results for each time point (from all 4 participants) during the 8‐h study presented as a time profile. (b) is a graphical representation of the mean serum FSH over the study period shown in (a). Black line and bar, vehicle; green line and bar, kisspeptin‐54 0·1 nmol/kg/h; blue line and bar, kisspeptin‐54 0·3 nmol/kg/hr; red line and bar, kisspeptin‐54 1·0 nmol/kg/h. Data are mean ± SEM. *N* = 4/group. Green *, *P* < 0·05 for SC 0·1 nmol/kg/h kisspeptin‐54 *vs* vehicle; blue ***, *P* < 0·001 for SC kisspeptin‐54 0·3 nmol/kg/h *vs* vehicle; red ***, *P* < 0·001 for SC kisspeptin‐54 1·0 nmol/kg/h *vs* vehicle.

Fig. 3a shows the time profile of the study with mean FSH values for each time point from the 4 participants receiving the same treatment. There was a significant increase in serum FSH with all doses of SC kisspeptin‐54 when compared with vehicle (*P* < 0·05, 0·1 nmol/kg/h *vs* vehicle; *P* < 0·001, 0·3 nmol/kg/h *vs* vehicle; *P* < 0·001, 1·0 nmol/kg/h *vs* vehicle). Fig. 3b shows the mean serum FSH for each intervention over the study period from the data presented in Fig. 3a.

**Figure 4:**
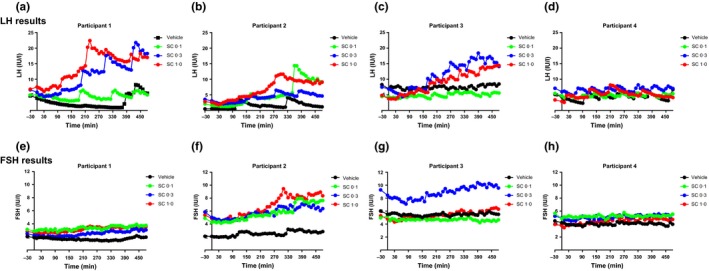
Individual effects on serum LH and FSH during 8‐h infusions of vehicle and SC kisspeptin‐54 for each participant. Individual time profiles from each participant 1–4 are presented showing the effects on serum LH (a–d) and FSH (e–h) during infusions of vehicle and SC kisspeptin‐54 0·1, 0·3 and 1·0 nmol/kg/h in healthy women during the early follicular phase of the menstrual cycle. Infusions started at *t* = 0 min and continued for 8‐h until *t* = 480 min, 10 minutely blood sampling was conducted from *t* = 0 min. Black circles, vehicle; green circles, kisspeptin‐54 0·1 nmol/kg/h; blue circles, kisspeptin‐54 0·3 nmol/kg/h; red circles, kisspeptin‐54 1·0 nmol/kg/h.

Fig. 4 shows the individual time profiles of the 4 participants receiving infusions of vehicle and SC kisspeptin‐54 at 3 doses.

LH pulsatility was formally analysed by a deconvolution method and although the number of LH pulses increases with increasing doses of kisspeptin‐54 (Fig. S1A), this difference did not reach statistical significance (number of LH pulses per 8‐h: 4·3 ± 1·1, vehicle; 5·5 ± 1·8 kisspeptin‐54 0·1 nmol/kg/h; 6·0 ± 1·3 kisspeptin‐54 0·3 nmol/kg/h; 6·0 ± 0·7 kisspeptin‐54 1·0 nmol/kg/h). The LH pulse amplitude (Fig. S1B) did not show any difference between the dosing groups (mean LH pulse amplitude in IU/l: 5·41 ± 0·89, vehicle; 8·10 ± 2·11 kisspeptin‐54 0·1 nmol/kg/h; 7·31 ± 1·67 kisspeptin‐54 0·3 nmol/kg/h; 6·92 ± 1·76 kisspeptin‐54 1·0 nmol/kg/h).

### Baseline oestradiol *vs* LH response to kisspeptin administration

Using regression analysis, the baseline oestradiol levels of each woman were correlated with her serum LH response to SC kisspeptin‐54 at the 3 doses tested. The results suggest there was a highly significant relationship between baseline oestradiol and mean LH for the 0·3 and 1·0 nmol/kg/h groups as shown in Table 2 (regression coefficients and *P*‐value for the change in mean LH for a 100 pmol/l rise in baseline oestradiol: −0·25, *P* = 0·26, vehicle; −1·75, *P* = 0·24, 0·1 nmol/kg/h; 0·83, *P* = 0·03, 0·3 nmol/kg/h; 1·00, *P* = 0·03, 1·0 nmol/kg/h). The positive regression coefficients imply that higher values of baseline oestradiol were associated with higher LH values in the 2 highest doses tested. For the 1·0 nmol/kg/h group, a 100pmol/l rise in baseline oestradiol was associated with a 1·0 IU/l increase in LH.

**Table 2:** Effects of baseline oestradiol on LH response to kisspeptin‐54 administration. Linear regression analysis was used to determine whether there was an association between baseline oestradiol and the LH response to SC kisspeptin‐54 at 3 doses (0·1, 0·3 and 1·0 nmol/kg/h) and vehicle, when administered to healthy women in the early follicular phase of the menstrual cycle. The regression coefficients and confidence intervals presented represent the change in mean LH for a 100pmol/l increase in baseline oestradiol

Group	Coefficient (95% CI)	*P*‐value
Vehicle	−0·25 (−0·72, 0·23)	0·26
0·1 nmol/kg/h kisspeptin‐54	−1·75 (−4·85, 1·36)	0·24
0·3 nmol/kg/h kisspeptin‐54	0·83 (0·12, 1·53)	0·03
1·0 nmol/kg/h kisspeptin‐54	1·00 (0·11, 1·89)	0·03

## Discussion

This proof of concept study shows that SC administration of kisspeptin is a viable option for future administration to stimulate gonadotrophin release in women. In this study, kisspeptin had a greater effect on LH compared to FSH release at the doses tested. This is consistent with previous studies in humans which show that kisspeptin administration has greatest effects on LH release with smaller effects on FSH release.^14^, ^19^ Testing higher doses of SC infusion of kisspeptin in the future would be useful to establish the full dosing range and efficacy of this approach for therapeutic potential.

Oestradiol may be an important determinant of LH response to kisspeptin. *In vitro* studies have shown that in GnRH neuronal cell lines oestradiol enhanced GnRH secretion in response to kisspeptin administration.^15^, ^16^ In hypothalamic ERα‐positive GT1‐7 cells, it was shown that oestradiol upregulated the expression of the kisspeptin gene.^22^ In mice, kisspeptin has been shown to stimulate GnRH neuronal activity and oestradiol further enhances kisspeptin‐induced GnRH neuronal activity.^23^ Guerriero *et al*. administered kisspeptin‐10 into the hypothalamus of pubertal female rhesus monkeys and directly measured GnRH production. They showed that ovariectomised pubertal rhesus monkeys not receiving oestradiol replacement produced little GnRH in response to kisspeptin when compared with ovariectomised and oestradiol replaced rhesus monkeys.^17^ The differences in response to kisspeptin over the ovulatory cycle in animals^24^, ^25^ and in humans^9^, ^14^, ^19^ may also be closely related to oestradiol levels. Oestradiol can both negatively and positively affect the axis depending on the phase of the menstrual cycle.^26^

These *in vitro* and *in vivo* animal studies suggest that oestradiol is an important determinant of LH response to kisspeptin. Thus, we compared baseline oestradiol levels with the LH response to SC kisspeptin infusion at 3 different doses. We showed that a kisspeptin infusion given at the 2 highest doses of 0·3 and 1·0 nmol/kg/h showed the greatest correlation between baseline oestradiol and LH response to kisspeptin. This suggested that at these doses the higher the baseline oestradiol, the better the response to kisspeptin. In fact, we have shown for the first time that a 100pmol/l rise in baseline oestradiol resulted in a 1·0 IU/l rise in LH in response to a SC infusion of 1·0 nmol/kg/h kisspeptin‐54.

During the menstrual cycle, the response to kisspeptin is greatest in the preovulatory phase when there are high oestradiol levels.^9^, ^14^, ^19^ Of note subject 1 had higher than average baseline oestradiol levels than would be expected for the early follicular phase of the menstrual cycle. Her levels were more in keeping with late follicular phase levels; however, she did not have an exaggerated response to kisspeptin‐54 that is usually seen in the late follicular, preovulatory phase of the menstrual cycle.^19^ Therefore, her oestradiol levels are likely to represent individual variation rather than another phase of the menstrual cycle.

In summary, our data show that SC administration of kisspeptin is a potential option for future administration to stimulate gonadotrophin release in women to treat reproductive disorders. In addition our data suggest that baseline oestradiol levels may be an important determinant of the gonadotrophin response to kisspeptin treatment in women in the follicular phase of the menstrual cycle. Thus, suggesting that if kisspeptin is to be used therapeutically that it may be beneficial to replace the low oestradiol levels of patients in order to obtain the optimal response from kisspeptin administration. However, further studies are required to establish the correct dose and duration of treatment using SC kisspeptin infusion that causes a rise in gonadotrophins and to confirm that oestradiol supplementation improves the response to kisspeptin.

## Conflict of Interest and financial disclosure

Nothing to declare.

## Supporting information


**Figure S1**: Effects of SC infusions of kisspeptin‐54 over 8 h on LH pulsatility in healthy women. Healthy women received 8 h SC infusions of kisspeptin‐54 at 3 doses (0·1, 0·3 and 1·0 nmol/kg/h) and vehicle, 10 minutely blood samples were taken for the duration of the study and the LH pulses were analysed by using a blinded deconvolution method. Graph A shows LH pulses and graph B LH pulse amplitude. Black bar, vehicle; green bar, kisspeptin‐54 0·1 nmol/kg/h; blue bar, kisspeptin‐54 0·3 nmol/kg/h; red bar, kisspeptin‐54 1·0 nmol/kg/h.
