# Supplementary figures and images for: Subcutaneous infusion of kisspeptin‐54 stimulates gonadotrophin release in women and the response correlates with basal oestradiol levels
Source: Clin Endocrinol (Oxf). 2015 Dec 17;84(6):939–45. doi: 10.1111/cen.12977 (PMC4914955; doi:10.1111/cen.12977)

# Supplemental figure 1

**A**

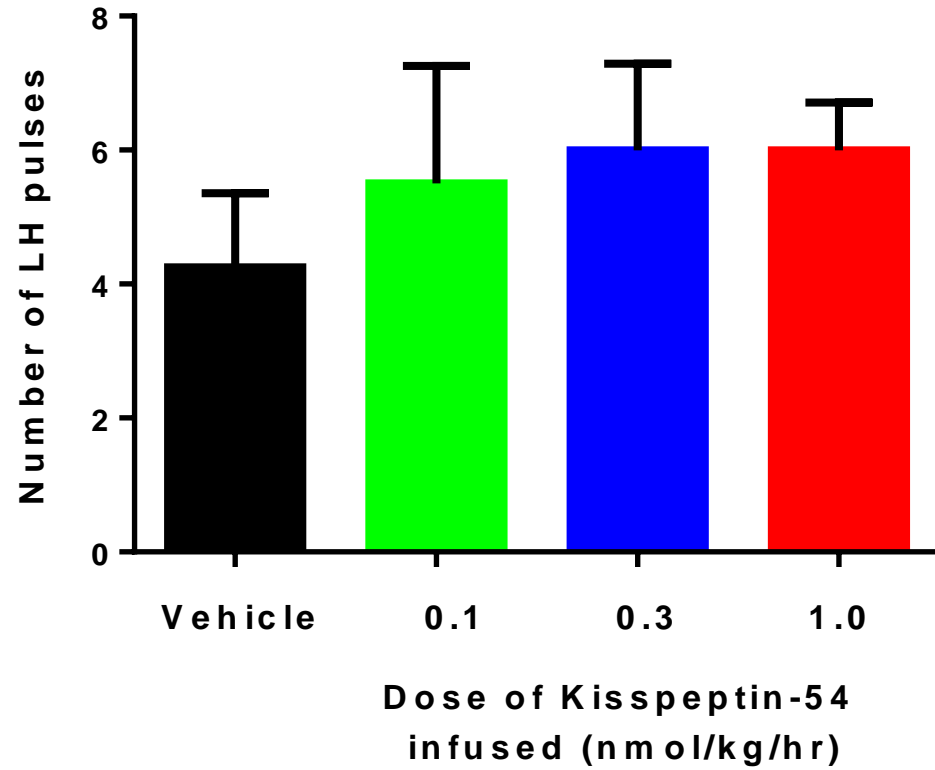

**B**

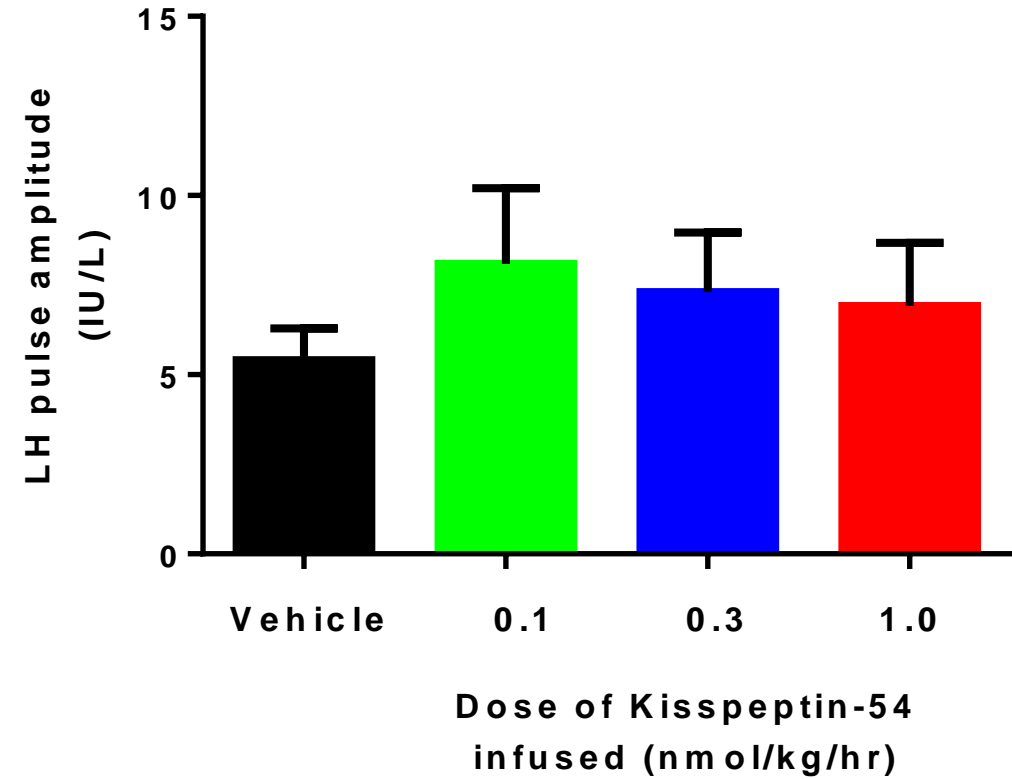

Supplement: Supplementary file 1 — Figure S1: Effects of SC infusions of kisspeptin‐54 over 8 h on LH pulsatility in healthy women. Healthy women received 8 h SC infusions of kisspeptin‐54 at 3 doses (0·1, 0·3 and 1·0 nmol/kg/h) and vehicle, 10 minutely blood samples were taken for the duration of the study and the LH pulses were analysed by using a blinded deconvolution method. Graph A shows LH pulses and graph B LH pulse amplitude. Black bar, vehicle; green bar, kisspeptin‐54 0·1 nmol/kg/h; blue bar, kisspeptin‐54 0·3 nmol/kg/h; red bar, kisspeptin‐54 1·0 nmol/kg/h. [file CEN-84-939-s001.pdf]
